# Exploring the Chemical Fingerprint and In Vitro Biocompatibility of Wild‐Growing and Ex Situ Cultivated *Stachys cretica* subsp. *cretica* in the Same Region (Crete, Greece)

**DOI:** 10.1002/cbdv.202503054

**Published:** 2026-01-06

**Authors:** Ekaterina‐Michaela Tomou, Maria Anagnostou, Francesco Cesare Battisti, Anastasia Karioti, Nikos Krigas, Nefeli Lagopati, Helen Skaltsa

**Affiliations:** ^1^ Department of Pharmacy Section of Pharmacognosy and Chemistry of Natural Products School of Health Sciences National and Kapodistrian University of Athens Athens Greece; ^2^ Laboratory of Pharmacognosy School of Pharmacy Aristotle University of Thessaloniki University Campus Thessaloniki Greece; ^3^ Department of Pharmacy University of Pisa Pisa Italy; ^4^ Institute of Plant Breeding and Genetic Resources Hellenic Agricultural Organization Demeter (ELGO‐Dimitra) Thermi Greece; ^5^ Department of Viticulture Floriculture & Plant Protection Institute of Olive Tree Subtropical Crops and Viticulture Hellenic Agricultural Organization‐Demeter (ELGO‐Dimitra) Athens Greece; ^6^ Department of Basic Medical Sciences Laboratory of Biology Medical School National and Kapodistrian University of Athens Greece; ^7^ Biomedical Research Foundation Academy of Athens Athens Greece

**Keywords:** cytotoxicity, HPLC‐PDA‐MS, Lamiaceae, natural products, NMR

## Abstract

The genus *Stachys* (Lamiaceae) comprises numerous species recognized for their ethnopharmacological importance and rich chemical profiles. In this study, we investigated the chemical composition and biocompatibility of both wild‐growing Cretan samples and asexually propagated samples of *Stachys cretica* L. subsp. *cretica* that were ex situ cultivated in the same region (Crete). The methanol (MeOH) extracts and infusions were analyzed using high‐performance liquid chromatography‐photodiode array detection coupled with mass spectrometry (HPLC‐PDA‐MS) and proton nuclear magnetic resonance (^1^H‐NMR) spectroscopy. A total of 23 compounds were identified in the analyzed samples by HPLC‐PDA‐MS, classified as flavonoids, phenylethanoid glycosides, and phenolic acids. The NMR screening confirmed the presence of chlorogenic acid, acteoside, lavandulifolioside, and leucosceptoside A in the MeOH extract of wild‐growing *S. cretica* subsp. *cretica*. Methanol extracts exhibited higher chemical diversity compared to infusions, particularly in flavonoids. Moreover, biocompatibility is crucial for most biomedical and pharmaceutical applications, ensuring that drugs, drug delivery systems, and cosmetics avoid possible side effects or cytotoxicity. Thus, normal human embryonic kidney cells (HEK293) were cultivated and cell viability (%) was estimated. This confirmed that *S. cretica* subsp. *cretica* extracts are biocompatible. The limited phytochemical variation observed between wild‐growing and cultivated samples suggests that cultivation within the species’ native range may preserve its chemical composition.

## Graphical abstract

Methanol extracts and infusions of wild and cultivated *Stachys cretica* subsp. *cretica* share similar profiles.

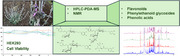

## 1 Introduction

The genus *Stachys* L. includes approximately 370 species (or 435 species and subspecies) and is recognized as one of the largest genera in the Lamiaceae family [1], predominantly found in the world's warm temperate zones, the Mediterranean, and Southwest Asia. Its distribution also extends to secondary regions in North and South America, along with southern Africa [2]. Most *Stachys* species thrive in diverse habitats, including forests, rocky areas, and limestone environments, and they can be classified as annual or perennial herbs, as well as small shrubs [3]. In Greece, there are 54 *Stachys* taxa (species and subspecies), with a notable proportion being local endemics confined to the region [4]. According to Bhattacharjee's classification (1980) [5], the genus *Stachys* is divided into two subgenera, *Stachys* and *Betonica*, with the former consisting of 19 sections and the latter containing two. In this classification, *Stachys cretica* L. subsp. *cretica* is placed in the section *Eriostomum*. This subspecies is a Steno‐Mediterranean entity primarily found in the eastern Mediterranean region, spanning from Krym (Transcaucasus) and Turkey‐in‐Europe to the Balkan Peninsula (including mainland and insular Greece, Bulgaria, and Albania), as well as Cyprus [1].

Different preparations from various Lamiaceae plants, including those of the genus *Stachys*, have been used in traditional medicine worldwide to treat infections, gastrointestinal disorders, inflammation, skin diseases, and respiratory conditions such as asthma [6, 7]. Moreover, phytochemical studies have revealed that *Stachys* species contain a variety of bioactive compounds, including terpenoids, iridoids, phytosterols, and polyphenols (such as flavonoids), among others [8, 9, 10]. These compounds are associated with numerous pharmacological effects, including antioxidant, anti‐inflammatory, anti‐diabetic, antimicrobial, and cytotoxic activities [8, 9].

Growing attention has been given to the antioxidant and anti‐inflammatory properties of *Stachys* species, particularly for their relevance in aging‐related processes [11, 12]. Studies on *Stachys* extracts have shown protective effects against cellular damage, supporting their potential role in promoting healthy aging [12, 13]. In continuation of our research on *Stachys* spp. [14, 15, 16, 17], the present study aimed to investigate the biocompatibility of *S. cretica* subsp. *cretica*, avoiding any possible renal toxicity. Biocompatibility is considered very crucial for the design and development of a great variety of biomedical and pharmaceutical applications, such as drug delivery systems or cosmetics [18]. To evaluate cytotoxicity, several methods are feasible. In this study, normal human embryonic kidney cells (HEK293) were cultivated to be used for the estimation of cell viability (%), employing MTT (3‐[4,5‐dimethylthiazol‐2‐yl]‐2,5 diphenyl tetrazolium bromide) colorimetric assay and thus ensuring that *S. cretica* subsp. *cretica* extracts are biocompatible without any effect on cell proliferation or viability.

## 2 Results and Discussion

### 2.1 High‐Performance Liquid Chromatography‐Photodiode Array Detection Coupled With Mass Spectrometry and Nuclear Magnetic Resonance Analyses

The liquid chromatography‐mass spectrometry (LC‐MS) profiling of wild‐growing and cultivated *S. cretica* subsp. *cretica* samples (Table 1) revealed notable chemical diversity between the infusions and the methanol (MeOH) extracts, which can be attributed to the different extraction methods and solvents used [19]. Specifically, the MeOH extracts (wild plants SCWM; cultivated plants SCCM) were richer in chemical compounds, particularly phenylethanoid glycosides and flavonoids, compared to the infusion samples (wild plants SCWI; cultivated plants SCCI). Similarly, a phytochemical analysis of the aqueous and MeOH extracts of *S. cretica* subsp. *anatolica* Rech.f. showed distinct differences in their chemical profiles, with the MeOH extract displaying a notably richer composition than the aqueous extract [20]. This may be explained by the fact that MeOH, as a solvent, can extract less polar constituents more effectively than water. As LC‐MS is aimed mainly at the identification of compounds, quantitative comparison between wild and cultivated samples cannot be reliably made.

**TABLE 1:** Fragmentation data (in negative mode) for the compounds detected in infusions and methanol extracts of wild‐growing (SCWI; SCWM) and cultivated (SCCI; SCCM) plant samples of *Stachys cretica* subsp. *cretica*.

No.	Rt (min)	UV (nm)	Negative mode, *m/z* (rel. intensity 100%)	Identification	Infusions		Methanol extracts	
					SCWI	SCCI	SCWM	SCCM
**1**	6.27	296, 325	191.1 [quinic acid‐H]‐, 353.1 [M‐H]‐	Chlorogenic acid	+	+	+	+
**2**	14.03	289, 331	137.1, 593.2 [M‐caffeoyl‐H]‐, 755.1 [M‐H]‐	Lavandulifolioside	+	+	+	+
**3**	14.85	289, 330	160.9 [caffeoyl group‐H]‐, 461.1 [M‐caffeoyl‐H]‐, 623.1 [M‐H]‐	Acteoside	+	+	+	+
**4**	15.99	252, 267, 346	285 [A‐H]‐, 447 [M‐H]‐	Luteolin‐7‐*O‐*glucoside	+	—	+	+
**5**	16.53	288, 327	137.1, 160.9 [caffeoyl group‐H]‐, 461.1 [M‐caffeoyl‐H]‐, 623.2 [M‐H]‐	Isoacteoside	—	—	+	—
6	17.48	251, 268, 336	299.1, 461.1, 623.1[M‐H]‐	Methoxy apigenin‐dihexoside	—	—	+	—
**7**	18.48	286, 328	769 [M‐H]‐	Alyssonoside	+	+	+	+
**8**	20.56	295, 331	637 [M‐H]‐	Leucosceptoside Α	+	+	+	+
**9**	23.00	252, 267, 348	299, 461	Chrysoeriol‐hexoside	+	—	—	—
**10**	21.26	268, 330	268.1 [A‐H, homolytic]‐, 431.1 [M‐H]‐	Apigenin‐glucoside	—	—	+	—
**11**	28.68	252, 267, 341	299, 665	Chrysoeriol‐acetyl‐dihexoside	—	—	+	+
**12**	29.37	275, 307, 314	285, [A‐H]‐, 651.1 [M‐H]‐	Isoscutellarein‐acetyl‐dihexoside	—	—	+	+
**13**	29.40	286, 333	175.1, 783.3	Stachysoside C, tentatively or Leucosceptoside B	—	—	+	—
**14**	29.84	253, 276, 300, 339	315, [A‐H]‐, 458.9 [M‐hexose‐acetyl‐H2O‐H]‐, 681.1 [M‐H]‐	3′‐Methylether‐hypolaetin‐acetyl‐dihexoside	—	—	+	+
**15**	30.52	253, 278, 302, 333	315, [A‐H]‐, 681.1 [M‐H]‐	3′‐Methylether‐hypolaetin‐acetyl‐dihexoside‐ isomer	—	—	—	+
**16**	31.07	288, 322	651.1 [M‐H]‐	Martynoside	—	—	+	+
**17**	39.31	269, 318	269, [A‐H]‐, 577.1 [M‐H]‐	Apigenin‐7‐coumaroyl‐hexoside	—	—	+	+
**18**	39.87	274, 315	269, [A‐H]‐, 577.1 [M‐H]‐	Apigenin‐coumaroyl‐hexoside isomer	—	—	+	+
**19**	40.55	285 ↑, 313	271, [A‐H]‐, 579.1 [M‐H]‐	Naringenin‐coumaroyl‐hexoside	—	—	+	+
**20**	40.98	284 ↑↑, 313	271, [A‐H]‐, 579.1 [M‐H]‐	Naringenin‐coumaroyl‐hexoside isomer	—	—	+	+
**21**	42.35	297, 307	342, 462, 582	Acylated spermine	—	—	+	+
**22**	43.04	270, 316	269, [A‐H]‐, 577.1 [M‐H]‐	Apigenin‐coumaroyl‐hexoside isomer	—	—	+	+
**23**	51.74	270, 315	269, [A‐H]‐, 593 [M‐coumaroyl residue‐H]‐, 723.1 [M‐H]‐	Apigenin‐dicoumaroyl‐hexoside isomer	—	—	+	+

The identified compounds are consistent with the phytochemical fingerprints typically reported for the genus *Stachys* [16]. In total, 23 compounds were detected across the four samples, with chlorogenic acid, lavandulifolioside, acteoside, leucosceptoside A, and alyssonoside being present in all.

Among phenolic acids, chlorogenic acid (**1**) was identified. This compound has also been previously reported in *S. recta* [21], *S. cretica* subsp. *smyrnaea* Rech.f. [22], and *S. iva* Griseb. (currently known as *S. horvaticii* Micevski) [14]. Notably, a comprehensive study by Karioti et al. [23] reported chlorogenic acid as the predominant phenolic compound across various Balkan *Stachys* species. Moreover, its extensive occurrence throughout the genus has been thoroughly documented [9]. Chlorogenic acid is a well‐studied compound with multiple pharmacological properties, including anti‐inflammatory, anti‐apoptotic, antihypertensive, antiviral, antitumor, antibacterial, and antioxidant activities [24]. Phenylethanoid glycosides are main compounds found within the genus *Stachys*, exhibiting notable chemotaxonomic relevance [8, 9]. Among them, acteoside (**3**) has been identified in several *Stachys* species, including *S. lanata* Crantz [25], *S. byzantina* K. Koch [26], and *S. germanica* L. subsp. *salviifolia* (Zen.) Gams [27]. Acteoside demonstrates a range of pharmacological activities, including strong anti‐inflammatory and antioxidant effects, neuroprotective properties, and the potential to inhibit cancer‐related pathways [28]. Additionally, leucosceptoside A (**8**) has been previously detected in other Greek native *Stachys* species such as *S. tetragona* Boiss. & Heldr. [29] and *S. iva* [14, 17] and is reported to possess notable anti‐inflammatory and antioxidant activities [30]. Lavandulifolioside (**2**) was first isolated from *S. lavandulifolia* Vahl [31] and has been reported in some other species, such as *S. macrantha* (C. Koch.) Stearn [32], *S. riederi* Cham. [33], and *S. iva* [14]. It has also been detected in traces (<1%) or small amounts in *S. recta* subsp. *recta* (synonym of *S. nitens* Janka), *S. beckeana* Dörfl. & Hayek, *S. zepcensis* Formánek, *S. alpina* L. subsp. *dinarica* Murb., and *S. plumosa* Griseb. or in relatively higher amounts (3.97%) in *S. atherocalyx* K. Koch [23]. Lavandulifolioside has been reported to possess cardiovascular activity and notable anti‑arrhythmic effects by modulating cardiac electrophysiology [34].

The genus *Stachys* constitutes a rich source of flavonoids [9]. Flavones, mainly their 7‐*O*‐acetylallosylglucosides and 7‐*O*‐glucosides, have been widely found within the subgenus *Stachys* [8, 9]. Among these, some derivatives of apigenin and chrysoeriol have previously been detected in wild‐growing *Stachys* taxa from Greece, such as apigenin and chrysoeriol 7‐*O*‐*β*‐D‐glucosides, as well as acylated forms such as 7‐(6″‐*E*‐*p*‐coumaroyl)‐D‐glucopyranosides and 7‐(3″‐*E*‐*p*‐coumaroyl)‐D‐glucopyranosides. In the case of apigenin, the corresponding *Z*‐isomers have also been identified to date. Additionally, 7‐*O*‐[6″‐*O*‐acetyl‐allosyl]‐(1→2)‐glucosides of both apigenin and chrysoeriol have been documented, as well as chrysoeriol 7‐*O*‐[6‴‐*O*‐acetyl‐allosyl]‐(1→2)‐glucoside [9]. In the present study, flavone derivatives were detected exclusively in MeOH extracts, with a similar flavonoid content observed in both the wild and cultivated samples. For example, luteolin‐7‐*O*‐glucoside (**4**), chrysoeriol‐hexosides (**9**), and isoscutellarein‐acetyl‐dihexoside (**12**) were identified in both MeOH samples, confirming their occurrence in members of this genus [9]. Notably, apigenin‐7‐glucoside (**10**) was detected only in the MeOH extract from the wild‐growing *S. cretica* subsp. *cretica*. Moreover, apigenin and naringenin *p*‐coumaroyl‐hexosides (**17** and **19**), along with their isomers (**18** and **20**), were also detected in both MeOH extracts of wild‐growing and the cultivated plant samples, aligning with previous reports on acylated flavone glucosides in *Stachys* spp. [9]. It is noteworthy that this is the first report of acylated spermine (**21**) in members of the genus *Stachys;* it is a polyamine, which activates defense responses in plants against both biotic and abiotic factors [35].

**FIGURE 1:**
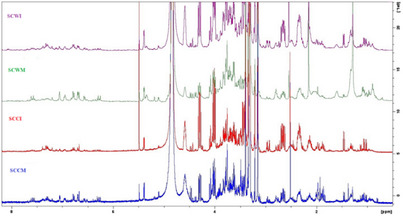
Overlaid proton nuclear magnetic resonance (^1^H‐NMR) spectra of infusions and methanol extracts from wild‐growing (SCWI; SCWM) and cultivated (SCCI; SCCM) plant material of *Stachys cretica* subsp. *cretica*.

Following LC‐MS analysis, proton nuclear magnetic resonance (^1^H‐NMR) screening of the *Stachys* samples was carried out, and the chemical categories of their components were identified based on peaks in specific regions. The overlaid ^1^H‐NMR spectra of the infusions and MeOH extracts (Figure 1) revealed variations in their chemical profiles, reflecting differences in metabolite composition among the samples, which could be attributed to the different extraction solvents and different methods of preparation [36].

All spectra generally displayed signals in the aliphatic (*δ* 0.5–3.0 ppm), sugar and oxygenated methylene (*δ* 3.0–5.5 ppm), and aromatic (*δ* 6.0–8.1 ppm) regions, indicative of the presence of constituents which could belong to terpenoids, flavonoids, phenolic compounds, and carbohydrates. Notably, the MeOH extracts displayed richer chemical profiles than the infusions, particularly in terms of flavonoids and phenolic acid derivatives (especially phenylethanoid glycosides), which is consistent with the LC‐MS results. Flavonoids and phelylethanoid glycosides are widely found in *Stachys* spp. [8, 9].

Regarding the comparison of ^1^H‐NMR spectra between cultivated and wild‐growing samples, the infusion from the cultivated sample (SCCI) exhibited a similar profile to that of the wild counterpart (SCWI). Likewise, the MeOH extract from the cultivated sample (SCCM) was close to the profile of the wild MeOH extract (SCWM).

**FIGURE 2:**
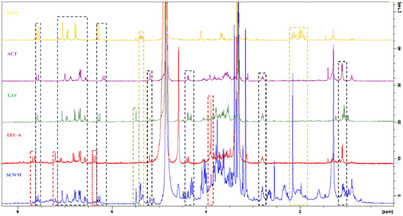
Overlaid proton nuclear magnetic resonance (^1^H‐NMR) spectra of wild‐growing *Stachys cretica* subsp. *cretica* methanol extract (SCWM) with previously isolated compounds leucosceptoside A (LEU‐A), lavandulofolioside (LAV), acteoside (ACT), and chlorogenic acid (CGA).

Based on prior LC‐MS analysis indicating that the MeOH sample of wild‐growing material (SCWM sample) contained slightly more constituents, this extract was selected for further NMR investigation. Its ^1^H‐NMR spectrum was compared to those of previously isolated compounds reported in our own earlier studies on *Stachys* spp. [14, 17]. Initially, chlorogenic acid was used to determine whether its signals could be identified in the ^1^H‐NMR spectrum of the SCWM sample, and its characteristic signals could be observed (Figure 2). This compound has been found in many *Stachys* spp. [16, 22, 23, 37].

As a next step, to interpret the signals corresponding to phenylethanoid glycosides, we compared the spectra with those of compounds that have been commonly found in *Stachys* spp. [8, 9] and have been previously isolated, namely acteoside, lavandulifolioside, and leucosceptoside A. The presence of these compounds was also supported by the afore‐mentioned LC‐MS results. The overlaid ^1^H‐NMR spectra of the SCWM extract and the reference compounds (chlorogenic acid, acteoside, lavandulifolioside, and leucosceptoside A) are shown in Figure 2, where key signals attributed to each compound are highlighted with differently coloured signals in the MeOH extract. It should be noted that alyssonoside was not included among the reference compounds; therefore, its presence could not be determined in the NMR analysis performed.

In addition, the ^1^H‐NMR spectrum of the SCWM was examined for the presence of flavonoids. Considering that isoscutellarein and hypolaetin derivatives are the main constituents in several *Stachys* taxa [8, 9], a comparison was made with previously isolated reference compounds. However, it was not feasible to fully identify their derivatives due to signal overlapping and their low concentration.

### 2.2 Cell Viability

Numerous studies have highlighted the broad spectrum of pharmacological properties exhibited by several members of the genus *Stachys*, with particular emphasis on their antioxidant and anti‐inflammatory activities [12]. These bioactive effects are considered fundamental mechanisms through which *Stachys* spp. may exert anti‐aging benefits [11]. The members of this genus are notably rich in secondary metabolites, including polyphenols, terpenoids, flavonoids, iridoids, and phenolic acids, which play pivotal roles in mitigating oxidative stress, a key contributor to cellular aging and degenerative diseases [9, 38]. Previous findings have demonstrated significant antioxidant capacity in both extracts and essential oils derived from various *Stachys* species, underscoring their potential in protecting biomolecular structures against oxidative damage associated with aging processes [12, 13]. Moreover, chronic low‐grade inflammation, recognized as a central feature of aging and a precursor to numerous age‐related pathologies, may be attenuated by the anti‐inflammatory properties of *Stachys* members [12, 13]. The anti‐inflammatory effects are primarily attributed to the modulation of pro‐inflammatory pathways by bioactive constituents such as flavonoids and iridoids, which have been shown to downregulate mediators of inflammation, thereby contributing to the prevention or delay of age‐associated disorders [9, 13]. All these desirable properties are related to the general biocompatibility of *Stachys* members [39]. It is of crucial importance to investigate any possible toxicity of a plant material before starting to develop a system or a drug that is going to be used for biomedical or even research purposes [40]. For example, possible toxicity to the kidneys, being organs that produce urine for excretion, should be avoided in every attempt to design a successful pharmaceutical approach [41]. Actually, biocompatibility of several materials with HEK293 cells is commonly assessed to determine their suitability for biomedical applications, and in tissue engineering and drug delivery systems development [42]. HEK293 cells are considered an in vitro model since they present characteristics like those of epithelial cells, and due to their high transfection efficiency [43]. The cells’ morphology, viability, and proliferation rate are employed in well‐established methods investigating the biological effects of either artificial or natural materials [43]. If any undesirable effect is detected on this type of cell in the presence of a factor, consequently, this means that this factor is not suitable for further investigation if its biocompatibility is the preferred characteristic depending on the application [44, 45].

**FIGURE 3:**
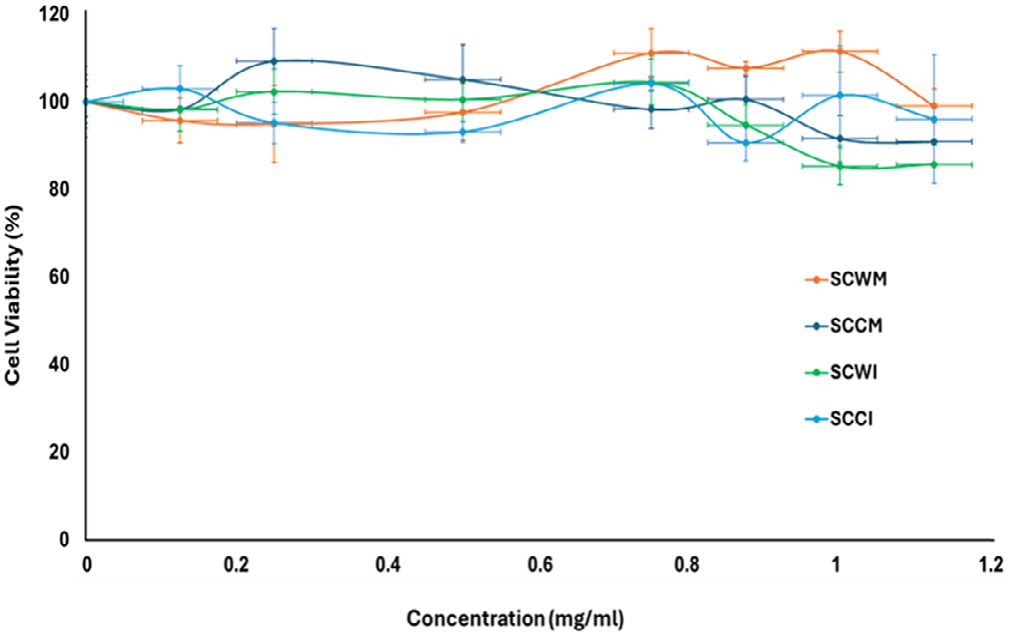
Effect of methanol extracts from wild (SCWM) and cultivated (SCCM) and infusions from wild (SCWI) and cultivated (SCCI) plant materials of *Stachys cretica* subsp. *cretica* on the viability of HEK293 (human embryonic kidney) cells. Data represents the standard deviation from three independent experiments.

Thus, in the present study, *S. cretica* subsp. *cretica* MeOH extracts and infusions of wild and cultivated plant materials were employed in testing on HEK293 cells using the MTT colorimetric assay and cell viability (%) was evaluated compared to untreated cells. There was no significant effect (*p*‐value = 0.1564) of any of these four samples on the viability of HEK293 cells, as it is presented in Figure 3 (*p* < 0.05 was considered statistically significant), applying the Kruskal–Wallis non‐parametric test for statistical analysis since the criteria for parametric ANOVA were not met. The normality was checked based on the Shapiro‐Wilk Test (α = 0.05). When running the SW test, the p‐value is 0.1765. Thus, Kruskal–Wallis was selected. These findings indicated that the tested extracts and infusions are non‐toxic to kidney cells. Even for concentrations over 0.8 mg/mL, HEK293 cell viability remains very high (>90%), so the samples can be considered biocompatible in this type of cells, ensuring that their use is safe in this range of concentrations. Those concentrations are considered extremely high, compared to conventional therapeutic doses that are typically a few µg/mL or ng/mL, so the fact that biocompatibility was indicated on kidney cells even in extremely higher doses is a very promising result. There is no significant difference compared to untreated cells (negative control), considering *p* < 0.05 versus negative control, based on the Kruskal‐Wallis non‐parametric test. These results showed that MeOH extracts and infusions of both wild and cultivated material of *S. cretica* subsp. *cretica* could be a promising candidate for further analysis aimed at biomedical applications.

## 3 Conclusions

This study provided a comprehensive chemical characterization of wild‐growing and cultivated plant material of *S. cretica* subsp. *cretica* originating from or growing in the same region (Crete), examining in the same period (spring) and in a comparative fashion, their MeOH extracts and infusions using HPLC‐PDA‐MS and NMR analyses. Methanol extracts were significantly more abundant in flavonoids and phenylethanoid glycosides compared to aqueous infusions, as demonstrated by LC‐MS analysis. The phenylethanoid glycosides, namely acteoside, lavandulifolioside, leucosceptoside A, and chlorogenic acid, in the MeOH extract of wild‐growing *S. cretica* subsp. *cretica* were also confirmed by the NMR analysis. The differences between wild‐growing and cultivated samples were of minor importance, thus suggesting that cultivation in the same region of origin may retain the authentic phytochemical species profile. Also, to avoid any toxicity of the samples, the MTT colorimetric assay was employed to investigate their effect on the viability of human embryonic kidney cells (HEK293) in vitro. The results obtained indicated that both the MeOH extracts and infusions from wild (SCWM and SCWI) and cultivated (SCCM, SCCI) plant materials of *S. cretica* subsp. *cretica* are biocompatible (cell viability), thus suitable for biomedical and pharmaceutical applications. Ongoing studies from our research group have shown that the extracts and infusions of *S. cretica* subsp. *cretica* are also non‐toxic to skin fibroblasts. Further thorough studies will shed light on the possible biocompatibility to other cell types.

## 4 Experimental

### 4.1 Plant Materials

**FIGURE 4:**
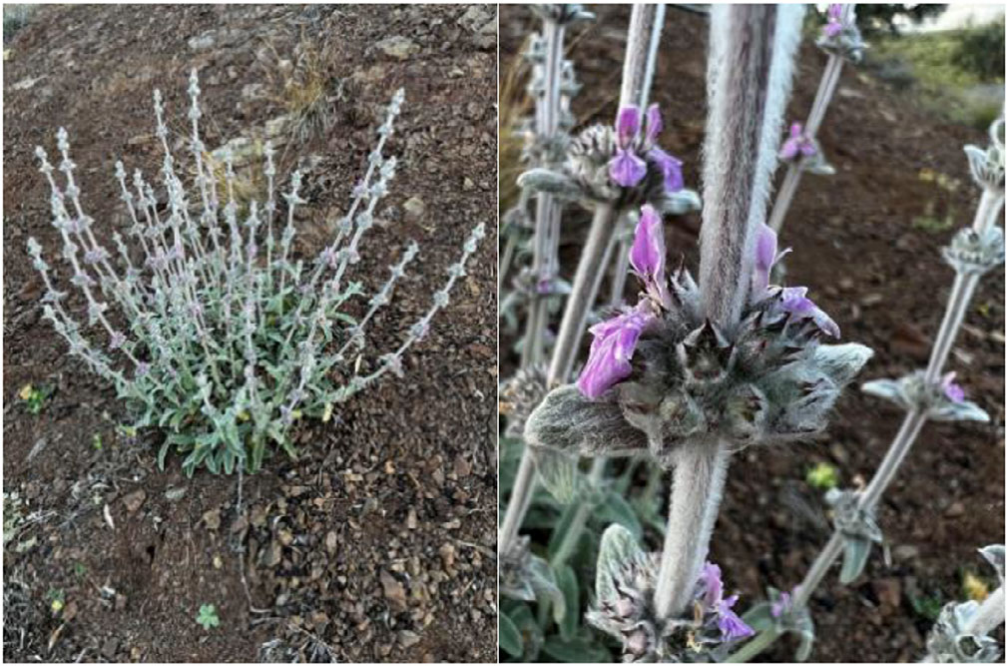
Aerial parts of a wild‐growing individual of *Stachys cretica* subsp. *cretica* (left) in Pateles, Ano Viannos, Crete (Greece) and a close‐up of the partial inflorescences arranged in whorls (right).

The wild‐growing populations of *S. cretica* subsp. *cretica* in Crete (Figure 4) were accessed and marked for photography and research using the special collection permit issued by the Greek Ministry of Environment and Energy (YPEN/DPD/15539/845 of 24/2/2022, YPEN/DPD/38262 /2306 of 2/8/2023, and YPEN/DPD/80381/5557 of 9/8/2024). Cuttings were excised in early March 2022 from wild‐growing *S. cretica* subsp. *cretica* at Pateles (N 35.01796, E 25.41825, 435 m above sea level) of Ano Viannos, Crete (Greece) for ex situ propagation at the premises of the Institute of Breeding and Plant Genetic Resources (IPBGR), Hellenic Agricultural Organization Demeter (ELGO‐Dimitra) using 0.2% indole‐3‐butyric acid (IBA; Racidin, Fytorgan ABEE, Greece) and were placed in a bench‐top fog system with an average air temperature of 19–25°C and relative humidity at 70%–85% for 20 days to allow rooting. The rooted cuttings were then transplanted into 0.33 L pots containing common soil, peat, perlite, and soft stones at 1:3:1:2 in a non‐heated greenhouse over winter 2022–2023, and were consequently transplanted into bigger (2.5 L) pots with the same substrate for late winter transfer and consequent cultivation in Crete (Adele, Rethymno) in late February 2023, where they were watered only a couple of times for acclimatization (completely rain fed onward, supervised by the external collaborator Evangelos Papiomytoglou). The aerial parts of the cultivated sample of S. *cretica* subsp. *cretica* were collected in 20/4/2023 from Adele, Rethymno, for comparative phytochemical analysis with the wild‐growing samples collected in 21/4/2023 during full flowering in Pateles, Ano Viannos (same origin as the cultivated sample, Figure 4). The plant samples were taxonomically authenticated by Dr N. Krigas, and a voucher specimen was deposited in the herbarium of Balkan Botanic Garden of Kroussia under the International Plant Exchange Network accession number GR‐1‐BBGK‐23,022 (GR‐1‐IPBGR‐23,022‐SCW for the wild‐growing material and GR‐1‐IPBGR‐23,022‐SCC for the asexually propagated and cultivated material of the same genotype).

### 4.2 Plant Preparations

The dried aerial parts of the wild‐growing *S. cretica* subsp. *cretica* (94 g) and the cultivated material of *S. cretica* subsp. *cretica* (148 g) were finely ground and macerated sequentially with dichloromethane (DCM) and then with MeOH (3 × 24 h per solvent). The resulting mixtures were filtered, and the solvents were evaporated under reduced pressure at low temperature using a rotary evaporator to yield dry residues: 206 mg and 203.3 mg for DCM extracts, as well as 207.1 mg and 200.3 mg for MeOH extracts.

In addition, 4 g finely comminuted aerial parts from each plant sample were infused in 200 mL of boiling water for 5 min, separately. Since *Stachys* infusions have traditionally been prepared and consumed in Greece as a kind of ‘mountain tea’, a term commonly associated with various members of the genus *Sideritis*, the infusion preparation followed the guidelines outlined in the European Medicines Agency monograph on different species and subspecies of the genus *Sideritis* [46]. Then, the samples were filtered, and the solvents were evaporated under reduced pressure at low temperature using a rotary evaporator to yield 2.4 g and 2.5 g, respectively.

### 4.3 HPLC‐PDA‐MS and NMR Analyses

The HPLC‐PDA‐MS analysis was conducted using a Thermo Finnigan system (Palo Alto, CA, USA), which included an LC Pump Plus, an Autosampler, and a Surveyor PDA Plus Detector. The system was coupled to an ESI MSQ Plus mass spectrometer (Thermo Finnigan, San Jose, CA, USA), controlled by Xcalibur software (version 2.1). The mass spectrometer operated in both positive and negative ionization modes, scanning within an m/z range of 100 to 1000. The gas temperature was set at 350°C, with a nitrogen flow rate of 10 L/min and a capillary voltage of 3000 V. The cone voltage ranged between 60 and 110 V. Chromatographic separation was carried out on an SB‐Aq Zorbax RP‐C18 column (Agilent, Santa Clara, CA, USA) measuring 150 mm × 3.5 mm, with a particle size of 3.5 µm, maintained at 30°C. The mobile phase consisted of water adjusted to pH 2.8 with 0.05% (v/v) formic acid (eluent A) and acetonitrile (eluent B), at a flow rate of 0.4 mL/min. The gradient program was structured as follows: 0–7 min, 90%–85% A; 7–12 min, 85%–82% A; 12–25 min, 82% A; 25–27 min, 82%–75% A; 27–32 min, 75% A; 32–42 min, 75%–60% A; 42–49 min, 60% A; 49–53 min, 60%–90% A; and 53–60 min, 90% A. The sample injection volume was 5 µL. Ultraviolet‐visible spectra were recorded over a wavelength range of 220–600 nm, while chromatographic profiles were monitored at 280, 330, and 350 nm. The identification of the compounds was based on co‐elution with the isolated compounds.

NMR spectra were acquired in CD_3_OD (for infusions and MeOH extracts) using a Bruker DRX 400 spectrometer operating at 399.95 MHz for ^1^H‐NMR. Chemical shifts (*δ*) were reported in ppm and referenced to the solvent signal at 3.31 ppm for CD_3_OD. Αll samples were stored in activated desiccators containing P_2_O_5_, ensuring the complete removal of moisture before the NMR analysis.

#### 4.3.1 Cell Culture

HEK293 from ATCC (Human embryonic kidney 293 cells; HEK293: HTB‐22TM) (LGC Standards GmbH, ATCC, Wesel, Germany) cells were cultured in Dulbecco's modified Eagle's medium (Gibco BRL, Life Technologies, ThermoScientific, Paisley, UK), adding 10% fetal bovine serum (FBS) and 1% antibiotics (penicillin/streptomycin) (Gibco BRL, Life Technologies, Thermo Scientific, Paisley, UK) to the medium, and the cells were maintained at 37°C under 5% CO_2_ [47, 48].

### 4.4 MTT Assay

The cytotoxicity of the *S. cretica* subsp. *cretica* extracts was estimated, applying MTT colorimetric assay (Thiazolyl Blue Tetrazolium Bromide M5655, Sigma‐Aldrich, Darmstadt, Germany). A spectrophotometer was utilized to quantify the cell viability by measuring the optical density of each of the tested samples. In total, 8000–10 000 cells/well were seeded in 96‐well plates and treated in increasing concentrations of each extract, ranging from 0 to 0.125 mg/mL. At 24 h post‐treatment, the medium was removed from each sample, and 10 µL of MTT solution (concentration 5 mg/mL in phosphate‐buffered saline (Gibco BRL, Life Technologies, ThermoScientific, Paisley, UK) was added. After 2 h of incubation, the supernatant was removed, and 100 µL of dimethyl sulfoxide was added to each well. The plates were left on a shaker for 30 min at room temperature, and right after, the optical density was measured at 570 nm (background normalization was achieved through one more measurement at 650 nm). Cell viability (%) was estimated compared to untreated cells. Applying the Kruskal–Wallis non‐parametric test for statistical analysis, *p* < 0.05 was considered statistically significant.

## Author Contributions

**Ekaterina‐Michaela Tomou**: methodology, validation, formal analysis, investigation, writing – original draft preparation, writing – review and editing, and visualization. **Maria Anagnostou**: validation, investigation, writing – original draft preparation, writing – review and editing, and visualization. **Francesco Cesare Battisti**: investigation. **Anastasia Karioti**: HPLC‐PDA‐MS analysis. **Nikos Krigas**: plant materials and writing – review and editing. **Nefeli Lagopati**: conceptualization, methodology, formal analysis, resources, writing – original draft preparation, writing – review and editing, visualization, and supervision. **Helen Skaltsa**: conceptualization, methodology, formal analysis, resources, writing – review and editing, and supervision. All authors have read and agreed to the published version of the manuscript.

## Conflicts of Interest

The authors declare no conflicts of interest.

## Funding

This research received no external funding.

## Data Availability

The authors have nothing to report.
